# *catena*-Poly[ammonium [aqua­bis­(μ-2,3,5,6-tetra­oxo-4-nitro­pyridin-4-ido)argentate(I)]]

**DOI:** 10.1107/S1600536813020631

**Published:** 2013-07-31

**Authors:** Nguyen Dinh Do, Olga Kovalchukova, Adam Stash, Svetlana Strashnova

**Affiliations:** a Hanoi University of Mining and Geology, Dong Ngac, Tu Liem, Ha Noi, Vietnam; b Peoples’ Friendship University of Russia, 6 Miklukho-Mallaya, 117198 Moscow, Russian Federation; c Karpov Institute of Physical Chemistry, 10 Vorontsovo Pole, 105064 Moscow, Russian Federation

## Abstract

In the title compound, {(NH_4_)[Ag(C_5_HN_2_O_6_)_2_(H_2_O)]}_*n*_, the Ag^I^ cation is seven-coordinated and is surrounded by four oxo O atoms of the 2,3,5,6-tetra­oxo-4-nitro­pyridin-4-ide species [Ag—O = 2.3848 (19), 2.4931 (18), 2.5361 (18) and 2.573 (2) Å], two nitro O atoms [Ag—O = 2.644 (2) and 2.661 (2) Å], and one water mol­ecule [Ag—O = 2.3133 (19) Å]. The pyridin-4-ide mono-anions act as polydentate bridging ligands and form a three-dimensional network that is stabilized through O—H⋯O and N—H⋯O hydrogen bonds involving the coordinating water mol­ecule and the imide function as donator groups. The ammonium cations are located in the cavities of the framework and are also involved in hydrogen bonding to O atoms of the ligand.

## Related literature

For reviews of 1,2-dicarbonyl compounds, see: Aldoshin (2008 ▶); Ohba & Okawa (2000 ▶). The synthesis and crystal structures of ammonium and sodium 2,3,5,6-tetra­oxo-4-nitro­pyridinates have been reported previously (Palkina *et al.*, 2000 ▶; Kuzmina *et al.*, 2004 ▶). The structure of the organic anion in its hexa­aqua metal salts is described by Kovalchukova *et al.* (2003 ▶ and 2013 ▶). For references to related structures of metal complexes with cyclic polyoxo compounds, see: Coronado *et al.* (2007 ▶); Kitagawa & Kawata (2002 ▶).
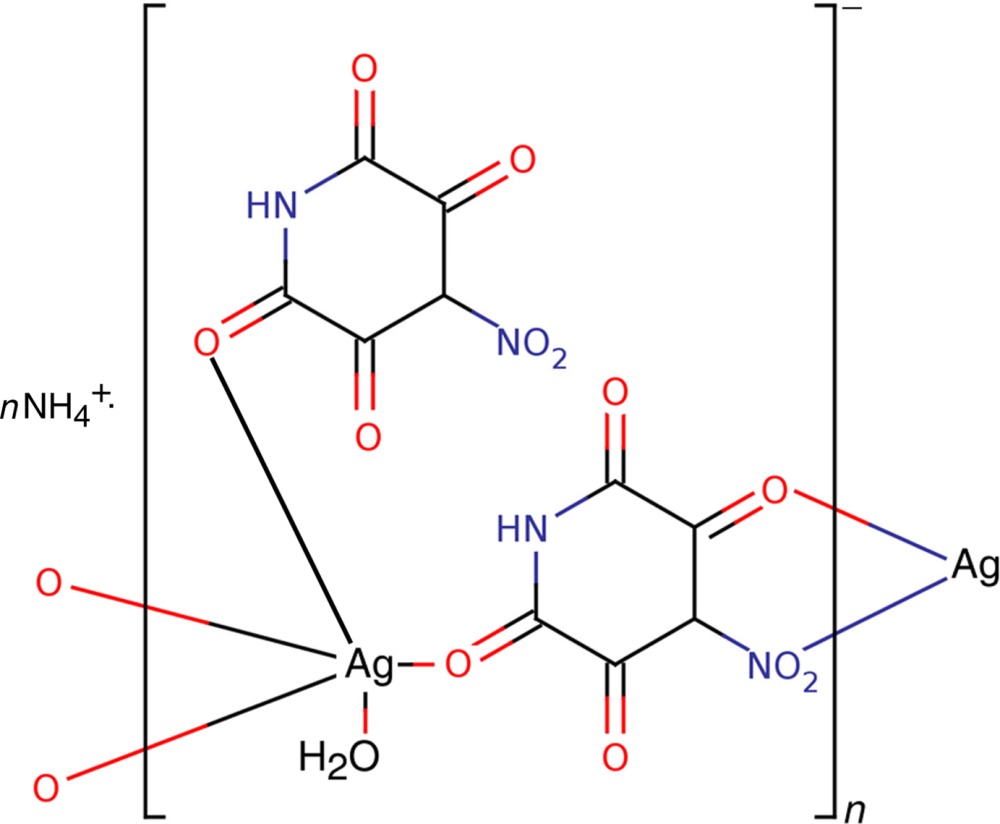


## Experimental

### 

#### Crystal data

(NH_4_)[Ag(C_5_HN_2_O_6_)_2_(H_2_O)]
*M* *_r_* = 514.08
Monoclinic, 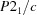

*a* = 8.784 (2) Å
*b* = 18.551 (4) Å
*c* = 9.195 (2) Å
β = 90.70 (3)°
*V* = 1498.2 (5) Å^3^
*Z* = 4
Mo *K*α radiation
μ = 1.44 mm^−1^
*T* = 293 K
0.35 × 0.31 × 0.08 mm

#### Data collection

Enraf Nonius CAD-4 diffractometer
Absorption correction: part of the refinement model (Δ*F*) (Walker & Stuart, 1983 ▶) *T*_min_ = 0.406, *T*_max_ = 0.798
2952 measured reflections
2768 independent reflections
2094 reflections with *I* > 2σ(*I*)
*R*_int_ = 0.014
3 standard reflections every 60 min intensity decay: none

#### Refinement

*R*[*F* ^2^ > 2σ(*F* ^2^)] = 0.021
*wR*(*F* ^2^) = 0.066
*S* = 1.09
2768 reflections
283 parameters
11 restraints
H atoms treated by a mixture of independent and constrained refinement
Δρ_max_ = 0.42 e Å^−3^
Δρ_min_ = −0.58 e Å^−3^

### 

Data collection: *CAD-4-PC* (Enraf–Nonius, 1993 ▶); cell refinement: *CAD-4-PC*; data reduction: *CAD-4-PC*; program(s) used to solve structure: *SHELXS97* (Sheldrick, 2008 ▶); program(s) used to refine structure: *SHELXL97* (Sheldrick, 2008 ▶); molecular graphics: *SHELXTL* (Sheldrick, 2008 ▶); software used to prepare material for publication: *CIFTAB97* and *SHELXL97*.

## Supplementary Material

Crystal structure: contains datablock(s) I, global. DOI: 10.1107/S1600536813020631/pj2004sup1.cif

Structure factors: contains datablock(s) I. DOI: 10.1107/S1600536813020631/pj2004Isup2.hkl

Additional supplementary materials:  crystallographic information; 3D view; checkCIF report

## Figures and Tables

**Table 1:** Hydrogen-bond geometry (Å, °)

*D*—H⋯*A*	*D*—H	H⋯*A*	*D*⋯*A*	*D*—H⋯*A*
N4—H4⋯O11^i^	0.86	2.18	2.979 (3)	155
N10—H10⋯O2	0.86	2.26	2.945 (3)	137
N10—H10⋯O3	0.86	2.29	3.030 (3)	144
O1—H11⋯O131^ii^	0.80 (3)	2.06 (3)	2.851 (3)	177 (5)
O1—H12⋯O8^iii^	0.80 (3)	2.02 (3)	2.781 (2)	160 (3)
N2—H21⋯O6^iv^	0.83 (2)	2.16 (2)	2.962 (3)	163 (3)
N2—H22⋯O72^v^	0.83 (2)	2.20 (2)	2.998 (3)	160 (3)

Symmetry codes: (i) 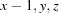; (ii) 
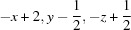
; (iii) 
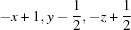
; (iv) 
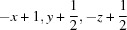
; (v) 
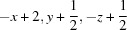
.

**Fig. 1.**
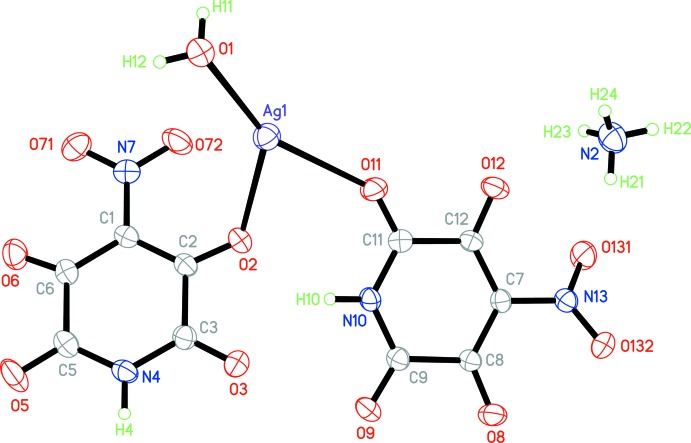
ORTEP view of (NH4)[Ag(C5HN2O6)2(H2O)] with atom labeling scheme (displacement ellipsoids are drawn at the 50% probability level for non-hydrogen atoms).

**Fig. 2.**
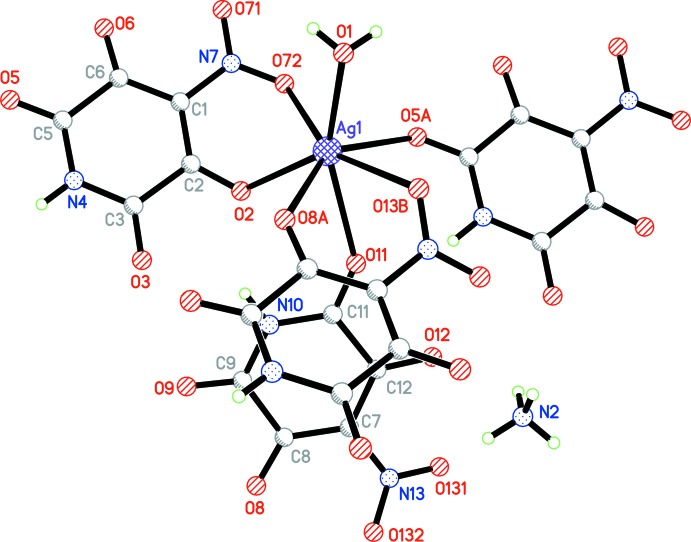
Structure of the coordination sphere of Ag(I).

**Fig. 3.**
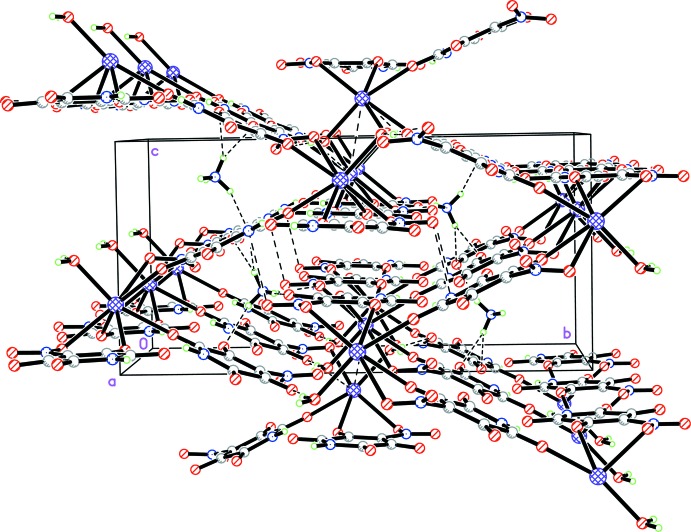
Molecular packing in the crystal of the complex along the crystallographic axis c.

## supplementary crystallographic information

### Comment

1,2-dicarbonyl compounds attract great interest because of the features of their structure and high reactivity. One of the simplest representatives of them is oxalic acid. As described by S. Aldoshin (Aldoshin, 2008), bimetallic coordination polymers based on oxalate and thiooxalate bridging ligands possess different types of magnetic activity and can intercalate complex organic molecules and ions. These have been extensively used as building units in supramolecular coordination systems (Ohba & Okawa, 2000). The replacement of oxalate anions by other 1,2-dicarbonyl cyclic compounds may be of interest from the synthetic and practical point of view. As an example (Coronado *et al.* 2007), the paramagnetic and chiral anion [Fe(C_5_O_5_)_3_]^3-^ has been combined with the organic donor BEDT-TTF DET (bis(ethylenedithio)tetrathiafulvalene) to synthesize a novel paramagnetic semiconductor with the first chirality-induced α-phase, α-(BEDT-TTF)5[Fe(C_5_O_5_)_3_].5H_2_O, and one of the few known paramagnetic molecular metals, β-(BEDT-TTF)5[Fe(C_5_O_5_)_3_].C_6_H_5_CN. The variety of coordination modes, some geometric characteristics as well as electrical, magnetic and other properties of coordinate compounds of dibenzoquinone-1,4 derivatives of a general formula H_2_C_6_O_4_*X*_2_ are summarised by S. Kitagawa and S. Kawata (Kitagawa & Kawata, 2002). The present paper deals with the crystal structure determination of ammonium-silver 2,3,5,6-tetraoxo-4-nitropyridinate monohydrate (NH_4_)[Ag(C_5_HN_2_O_6_)_2_(H_2_O)]. The molecular structure of the above substance consists of Ag(I) and ammonium cations, two crystallographically unequivalent 2,3,5,6-tetraoxo-4-nitropyridinate mono anions, and one coordinated water molecule. Each of the Ag(I) cation displays sevenfold coordination by O2, O5, O8, and O11 of the keto-groups of the organic species. The Ag—O distances are 2.3848 (19); 2.4931 (18); 2.5361 (18); and 2.573 (2) Å. Two coordinate bonds involve the O atoms of the nitro-group of the organic anion (2.644 (2) and 2.661 (2) Å). The shortest distance in the coordination sphere of Ag(I) involves the coordinated water molecule (2.3133 (19) Å). The 2,3,5,6-tetraoxo-4-nitropyridinate anions act as polydentate bridging ligands. This coordination mode leads to formation of polymer chains. The coordination does not change significantly the C—O distances of the ligand comparing with its ammonium and sodium salts (Palkina *et al.*, 2000; Kuzmina *et al.*, 2004). The corresponding bond lengths are in the range 1.224 (3) to 1.220 (3) Å for the nitro-diketone fragment, and 1.210 (4) to 1.215 (4) Å for the amide fragment. These two fragments of the organic mono anion are connected by an almost single C—C bonds (C2—C3 length is 1.531 (4), and C5—C6 length is 1.544 (4) Å). The ammonium cation has the outer sphere character, and forms bridging H-bonds with the O atoms of the organic anions linking the polymer chains into three-dimensional structure. The H atoms of the coordinated water molecules are also involved into the H-bonding.

### Experimental

Single crystals of C_10_H_8_AgN_5_O_13_ were grown by the slow evaporation of the ethanol solution of the 1-to-1 molar mixture of silver nitrate and ammonium 2,3,5,6-tetraoxo-4-nitropyridinate.

### Refinement

The structure of of (NH_4_)[Ag(C_5_HN_2_O_6_)_2_(H_2_O)] was solved by direct method and all non-hydrogen atoms were located and refined in anisotropically. All the hydrogen atoms were located in difference electron density syntheses and their positions refined subject to chemically reasonable restraints.

### Crystal data

**Table:**

(NH_4_)[Ag(C_5_HN_2_O_6_)_2_(H_2_O)]	*F*(000) = 1016
*M**_r_* = 514.08	*D*_x_ = 2.279 Mg m^−^^3^
Monoclinic, *P*2_1_/*c*	Mo *K*α radiation, λ = 0.71073 Å
Hall symbol: -P 2ybc	Cell parameters from 25 reflections
*a* = 8.784 (2) Å	θ = 9.3–11.8°
*b* = 18.551 (4) Å	µ = 1.44 mm^−^^1^
*c* = 9.195 (2) Å	*T* = 293 K
β = 90.70 (3)°	Plate, dark yellow
*V* = 1498.2 (5) Å^3^	0.35 × 0.31 × 0.08 mm
*Z* = 4	

### Data collection

**Table:**

Enraf Nonius CAD-4 diffractometer	2094 reflections with *I* > 2σ(*I*)
Radiation source: fine-focus sealed tube	*R*_int_ = 0.014
β-filter monochromator	θ_max_ = 25.5°, θ_min_ = 2.2°
ω/2θ scans	*h* = 0→10
Absorption correction: part of the refinement model (Δ*F*) Walker & Stuart (1983)	*k* = 0→22
*T*_min_ = 0.406, *T*_max_ = 0.798	*l* = −11→11
2952 measured reflections	3 standard reflections every 60 min
2768 independent reflections	intensity decay: none

### Refinement

**Table:**

Refinement on *F*^2^	Primary atom site location: structure-invariant direct methods
Least-squares matrix: full	Secondary atom site location: difference Fourier map
*R*[*F*^2^ > 2σ(*F*^2^)] = 0.021	Hydrogen site location: difference Fourier map
*wR*(*F*^2^) = 0.066	H atoms treated by a mixture of independent and constrained refinement
*S* = 1.09	*w* = 1/[σ^2^(*F*_o_^2^) + (0.0451*P*)^2^ + 0.0506*P*] where *P* = (*F*_o_^2^ + 2*F*_c_^2^)/3
2768 reflections	(Δ/σ)_max_ = 0.001
283 parameters	Δρ_max_ = 0.42 e Å^−^^3^
11 restraints	Δρ_min_ = −0.58 e Å^−^^3^

### Special details

**Table:**

Geometry. All e.s.d.'s (except the e.s.d. in the dihedral angle between two l.s. planes) are estimated using the full covariance matrix. The cell e.s.d.'s are taken into account individually in the estimation of e.s.d.'s in distances, angles and torsion angles; correlations between e.s.d.'s in cell parameters are only used when they are defined by crystal symmetry. An approximate (isotropic) treatment of cell e.s.d.'s is used for estimating e.s.d.'s involving l.s. planes.
Refinement. Refinement of *F*^2^ against ALL reflections. The weighted *R*-factor *wR* and goodness of fit *S* are based on *F*^2^, conventional *R*-factors *R* are based on *F*, with *F* set to zero for negative *F*^2^. The threshold expression of *F*^2^ > σ(*F*^2^) is used only for calculating *R*-factors(gt) *etc*. and is not relevant to the choice of reflections for refinement. *R*-factors based on *F*^2^ are statistically about twice as large as those based on *F*, and *R*- factors based on ALL data will be even larger.

### Fractional atomic coordinates and isotropic or equivalent isotropic displacement parameters (Å^2^)

**Table:**

	*x*	*y*	*z*	*U*_iso_*/*U*_eq_	
Ag1	0.68914 (2)	0.518350 (10)	0.16959 (2)	0.03092 (9)	
O1	0.6981 (2)	0.43383 (10)	−0.0151 (2)	0.0367 (4)	
O2	0.49428 (18)	0.55092 (10)	0.3355 (2)	0.0337 (4)	
O3	0.2721 (2)	0.64390 (10)	0.3252 (3)	0.0437 (5)	
O5	−0.0852 (2)	0.46912 (11)	0.3204 (2)	0.0430 (5)	
O6	0.1206 (2)	0.36677 (10)	0.3468 (2)	0.0380 (4)	
O71	0.4081 (2)	0.33294 (10)	0.3308 (2)	0.0374 (4)	
O72	0.5781 (2)	0.41601 (11)	0.3380 (3)	0.0434 (5)	
O8	0.59699 (19)	0.87943 (10)	0.5161 (2)	0.0346 (4)	
O9	0.40503 (19)	0.77524 (10)	0.4454 (2)	0.0371 (4)	
O11	0.7861 (2)	0.62851 (10)	0.3053 (2)	0.0335 (4)	
O12	0.99467 (19)	0.72206 (10)	0.3830 (2)	0.0361 (4)	
O131	1.0495 (2)	0.83839 (10)	0.5427 (3)	0.0440 (5)	
O132	0.8913 (2)	0.92310 (9)	0.4915 (2)	0.0337 (4)	
N2	1.1853 (2)	0.79954 (14)	0.1619 (3)	0.0356 (5)	
N4	0.0902 (2)	0.55773 (12)	0.3224 (2)	0.0277 (4)	
H4	0.0200	0.5900	0.3174	0.033*	
N7	0.4426 (2)	0.39746 (11)	0.3354 (2)	0.0255 (4)	
N10	0.5921 (2)	0.70207 (10)	0.3659 (2)	0.0234 (4)	
H10	0.5263	0.6718	0.3324	0.028*	
N13	0.9224 (2)	0.85893 (11)	0.5015 (2)	0.0250 (4)	
C1	0.3262 (2)	0.45034 (14)	0.3364 (2)	0.0232 (5)	
C2	0.3668 (3)	0.52426 (12)	0.3337 (2)	0.0230 (4)	
C3	0.2389 (3)	0.58051 (13)	0.3276 (3)	0.0267 (5)	
C5	0.0468 (3)	0.48715 (13)	0.3246 (3)	0.0264 (5)	
C6	0.1700 (3)	0.42790 (13)	0.3368 (2)	0.0243 (5)	
C7	0.8114 (2)	0.80583 (12)	0.4653 (2)	0.0219 (4)	
C8	0.6544 (2)	0.82305 (12)	0.4741 (2)	0.0212 (4)	
C9	0.5389 (3)	0.76440 (13)	0.4277 (2)	0.0236 (5)	
C11	0.7427 (3)	0.68490 (12)	0.3543 (2)	0.0222 (4)	
C12	0.8630 (3)	0.74009 (12)	0.4048 (2)	0.0222 (4)	
H11	0.768 (3)	0.4067 (17)	−0.019 (5)	0.074 (6)*	
H12	0.624 (3)	0.4089 (17)	−0.015 (5)	0.074 (6)*	
H21	1.109 (3)	0.8260 (14)	0.166 (4)	0.074 (6)*	
H22	1.263 (3)	0.8246 (14)	0.176 (4)	0.074 (6)*	
H23	1.182 (4)	0.7675 (14)	0.225 (3)	0.074 (6)*	
H24	1.190 (4)	0.7813 (16)	0.080 (2)	0.074 (6)*	

### Atomic displacement parameters (Å^2^)

**Table:**

	*U*^11^	*U*^22^	*U*^33^	*U*^12^	*U*^13^	*U*^23^
Ag1	0.03248 (13)	0.03008 (13)	0.03023 (13)	−0.00021 (7)	0.00124 (8)	−0.00114 (8)
O1	0.0281 (9)	0.0371 (10)	0.0449 (11)	−0.0023 (8)	0.0016 (8)	−0.0077 (8)
O2	0.0202 (8)	0.0337 (9)	0.0471 (11)	−0.0027 (7)	0.0010 (7)	−0.0058 (8)
O3	0.0299 (9)	0.0264 (9)	0.0747 (15)	0.0000 (8)	−0.0052 (9)	−0.0102 (9)
O5	0.0197 (9)	0.0485 (11)	0.0607 (13)	−0.0068 (8)	−0.0051 (8)	0.0142 (10)
O6	0.0330 (9)	0.0325 (10)	0.0485 (11)	−0.0067 (7)	−0.0014 (8)	0.0053 (8)
O71	0.0462 (11)	0.0284 (9)	0.0374 (10)	0.0044 (8)	−0.0032 (8)	0.0019 (8)
O72	0.0233 (9)	0.0408 (10)	0.0662 (14)	0.0076 (8)	0.0017 (9)	0.0079 (10)
O8	0.0241 (8)	0.0340 (9)	0.0455 (11)	0.0061 (7)	−0.0065 (8)	−0.0150 (8)
O9	0.0189 (9)	0.0374 (10)	0.0551 (12)	−0.0008 (7)	0.0040 (8)	−0.0096 (9)
O11	0.0280 (9)	0.0327 (10)	0.0398 (10)	0.0029 (7)	−0.0013 (7)	−0.0140 (8)
O12	0.0204 (9)	0.0394 (10)	0.0484 (11)	0.0035 (7)	0.0034 (8)	−0.0105 (8)
O131	0.0251 (9)	0.0359 (10)	0.0707 (14)	−0.0020 (8)	−0.0166 (9)	−0.0045 (9)
O132	0.0338 (9)	0.0223 (9)	0.0451 (11)	−0.0026 (7)	0.0010 (8)	−0.0044 (7)
N2	0.0285 (11)	0.0406 (13)	0.0376 (13)	−0.0039 (9)	−0.0015 (9)	−0.0011 (10)
N4	0.0200 (9)	0.0316 (10)	0.0313 (11)	0.0040 (8)	−0.0017 (8)	0.0003 (9)
N7	0.0291 (10)	0.0305 (11)	0.0168 (9)	0.0042 (8)	−0.0014 (7)	0.0022 (8)
N10	0.0193 (9)	0.0257 (10)	0.0253 (10)	−0.0024 (7)	−0.0025 (7)	−0.0033 (8)
N13	0.0232 (9)	0.0284 (10)	0.0234 (10)	−0.0014 (8)	−0.0005 (7)	−0.0030 (8)
C1	0.0214 (11)	0.0304 (12)	0.0178 (11)	0.0023 (9)	−0.0015 (8)	−0.0002 (9)
C2	0.0195 (10)	0.0311 (12)	0.0183 (10)	−0.0002 (9)	0.0004 (8)	−0.0034 (9)
C3	0.0244 (11)	0.0291 (13)	0.0266 (12)	−0.0007 (10)	−0.0020 (9)	−0.0059 (9)
C5	0.0234 (11)	0.0356 (13)	0.0202 (11)	−0.0018 (10)	−0.0018 (8)	0.0029 (10)
C6	0.0245 (11)	0.0300 (12)	0.0184 (11)	−0.0022 (9)	−0.0013 (9)	0.0011 (9)
C7	0.0211 (11)	0.0239 (10)	0.0204 (11)	−0.0023 (9)	−0.0028 (8)	0.0001 (9)
C8	0.0197 (10)	0.0250 (11)	0.0187 (10)	0.0006 (8)	−0.0022 (8)	−0.0022 (9)
C9	0.0221 (11)	0.0269 (11)	0.0218 (11)	−0.0003 (9)	−0.0013 (9)	−0.0024 (9)
C11	0.0250 (11)	0.0257 (11)	0.0157 (10)	0.0020 (9)	−0.0015 (8)	−0.0002 (9)
C12	0.0202 (11)	0.0257 (11)	0.0207 (10)	0.0020 (9)	0.0001 (8)	0.0005 (9)

### Geometric parameters (Å, º)

**Table:**

Ag1—O1	2.3133 (19)	O132—N13	1.225 (3)
Ag1—O2	2.3848 (19)	O132—Ag1^iv^	2.661 (2)
Ag1—O8^i^	2.4931 (18)	N2—H21	0.832 (19)
Ag1—O11	2.5361 (18)	N2—H22	0.83 (2)
Ag1—O5^ii^	2.573 (2)	N2—H23	0.830 (19)
Ag1—O72	2.644 (2)	N2—H24	0.827 (19)
Ag1—O132^i^	2.661 (2)	N4—C5	1.364 (3)
O1—H11	0.80 (3)	N4—C3	1.373 (3)
O1—H12	0.80 (3)	N4—H4	0.8600
O2—C2	1.224 (3)	N7—C1	1.417 (3)
O3—C3	1.212 (3)	N10—C11	1.366 (3)
O5—C5	1.206 (3)	N10—C9	1.373 (3)
O5—Ag1^iii^	2.573 (2)	N10—H10	0.8600
O6—C6	1.218 (3)	N13—C7	1.423 (3)
O71—N7	1.235 (3)	C1—C2	1.417 (3)
O72—N7	1.239 (3)	C1—C6	1.434 (3)
O8—C8	1.225 (3)	C2—C3	1.534 (3)
O8—Ag1^iv^	2.4931 (18)	C5—C6	1.546 (3)
O9—C9	1.206 (3)	C7—C12	1.417 (3)
O11—C11	1.203 (3)	C7—C8	1.419 (3)
O12—C12	1.223 (3)	C8—C9	1.544 (3)
O131—N13	1.235 (3)	C11—C12	1.539 (3)
			
O1—Ag1—O2	132.26 (6)	C5—N4—H4	117.9
O1—Ag1—O2	132.26 (6)	C3—N4—H4	117.9
O2—Ag1—O2	0.00 (5)	O71—N7—O72	120.3 (2)
O1—Ag1—O8^i^	96.52 (7)	O71—N7—C1	119.6 (2)
O2—Ag1—O8^i^	86.50 (7)	O72—N7—C1	120.0 (2)
O2—Ag1—O8^i^	86.50 (7)	C11—N10—C9	124.3 (2)
O1—Ag1—O11	153.27 (7)	C11—N10—H10	117.8
O2—Ag1—O11	73.76 (6)	C9—N10—H10	117.8
O2—Ag1—O11	73.76 (6)	O132—N13—O131	121.6 (2)
O8^i^—Ag1—O11	76.73 (7)	O132—N13—C7	120.24 (19)
O1—Ag1—O5^ii^	97.02 (7)	O131—N13—C7	118.21 (19)
O2—Ag1—O5^ii^	107.42 (7)	N7—C1—C2	119.23 (19)
O2—Ag1—O5^ii^	107.42 (7)	N7—C1—C6	119.3 (2)
O8^i^—Ag1—O5^ii^	144.87 (6)	C2—C1—C6	121.5 (2)
O11—Ag1—O5^ii^	76.64 (7)	O2—C2—O2	0.00 (16)
O1—Ag1—O72	87.67 (7)	O2—C2—C1	128.4 (2)
O2—Ag1—O72	62.25 (7)	O2—C2—C1	128.4 (2)
O2—Ag1—O72	62.25 (7)	O2—C2—C3	113.3 (2)
O8^i^—Ag1—O72	139.31 (6)	O2—C2—C3	113.3 (2)
O11—Ag1—O72	114.48 (7)	C1—C2—C3	118.3 (2)
O5^ii^—Ag1—O72	73.55 (6)	O3—C3—O3	0.0 (2)
O1—Ag1—O132^i^	78.24 (6)	O3—C3—N4	121.8 (2)
O2—Ag1—O132^i^	141.23 (6)	O3—C3—N4	121.8 (2)
O2—Ag1—O132^i^	141.23 (6)	O3—C3—C2	119.0 (2)
O8^i^—Ag1—O132^i^	63.63 (6)	O3—C3—C2	119.0 (2)
O11—Ag1—O132^i^	75.60 (6)	N4—C3—C2	119.2 (2)
O5^ii^—Ag1—O132^i^	87.76 (6)	O5—C5—N4	122.3 (2)
O72—Ag1—O132^i^	155.09 (5)	O5—C5—C6	118.5 (2)
Ag1—O1—H11	120 (3)	N4—C5—C6	119.2 (2)
Ag1—O1—H12	111 (3)	O6—C6—C1	127.8 (2)
H11—O1—H12	105.3 (9)	O6—C6—C5	114.7 (2)
O2—O2—C2	0 (10)	C1—C6—C5	117.6 (2)
O2—O2—Ag1	0 (6)	C12—C7—C8	122.1 (2)
C2—O2—Ag1	123.54 (16)	C12—C7—N13	117.8 (2)
O3—O3—C3	0 (10)	C8—C7—N13	119.65 (19)
C5—O5—Ag1^iii^	130.73 (17)	O8—C8—C7	127.9 (2)
N7—O72—Ag1	123.30 (16)	O8—C8—C9	114.66 (19)
C8—O8—Ag1^iv^	134.07 (15)	C7—C8—C9	117.45 (19)
C11—O11—Ag1	141.17 (16)	O9—C9—N10	122.3 (2)
N13—O132—Ag1^iv^	120.06 (15)	O9—C9—C8	118.9 (2)
H21—N2—H22	108.8 (9)	N10—C9—C8	118.83 (19)
H21—N2—H23	111 (3)	O11—C11—N10	122.9 (2)
H22—N2—H23	109.0 (9)	O11—C11—C12	118.2 (2)
H21—N2—H24	109.7 (9)	N10—C11—C12	118.9 (2)
H22—N2—H24	108 (3)	O12—C12—C7	127.4 (2)
H23—N2—H24	110.0 (9)	O12—C12—C11	114.6 (2)
C5—N4—C3	124.1 (2)	C7—C12—C11	118.00 (19)

Symmetry codes: (i) *x*, −*y*+3/2, *z*−1/2; (ii) *x*+1, *y*, *z*; (iii) *x*−1, *y*, *z*; (iv) *x*, −*y*+3/2, *z*+1/2.

### Hydrogen-bond geometry (Å, º)

**Table:**

*D*—H···*A*	*D*—H	H···*A*	*D*···*A*	*D*—H···*A*
N4—H4···O11^iii^	0.86	2.18	2.979 (3)	155
N10—H10···O2	0.86	2.26	2.945 (3)	137
N10—H10···O3	0.86	2.29	3.030 (3)	144
O1—H11···O131^v^	0.80 (3)	2.06 (3)	2.851 (3)	177 (5)
O1—H12···O8^vi^	0.80 (3)	2.02 (3)	2.781 (2)	160 (3)
N2—H21···O6^vii^	0.83 (2)	2.16 (2)	2.962 (3)	163 (3)
N2—H22···O72^viii^	0.83 (2)	2.20 (2)	2.998 (3)	160 (3)

Symmetry codes: (iii) *x*−1, *y*, *z*; (v) −*x*+2, *y*−1/2, −*z*+1/2; (vi) −*x*+1, *y*−1/2, −*z*+1/2; (vii) −*x*+1, *y*+1/2, −*z*+1/2; (viii) −*x*+2, *y*+1/2, −*z*+1/2.
